# Pupillary and Microsaccadic Responses to Cognitive Effort and Emotional Arousal During Complex Decision Making

**DOI:** 10.16910/jemr.13.5.2

**Published:** 2020-05-17

**Authors:** Krzysztof Krejtz, Justyna Żurawska, Andrew T. Duchowski, Szymon Wichary

**Affiliations:** Institute of Psychology, SWPS University of Social Sciences and Humanities, Warsaw, Poland; School of Computing, Clemson University, Clemson, SC, USA; Leiden Institute for Brain and Cognition, Leiden University, The Netherlands

**Keywords:** eye tracking, eye movement, microsaccades, pupillometry, decision making, emotional arousal, attention, cognitive effort

## Abstract

A large body of literature documents the sensitivity of pupil response to cognitive load (1)and emotional arousal (2). Recent empirical evidence also showed that microsaccade characteristics and dynamics can be modulated by mental fatigue and cognitive load (3). Very little is known about the sensitivity of microsaccadic characteristics to emotional arousal. The present paper demonstrates in a controlled experiment pupillary and microsaccadic responses to information processing during multi-attribute decision making under affective priming. Twenty-one psychology students were randomly assigned into three affective priming conditions (neutral, aversive, and erotic). Participants were tasked to make several discriminative decisions based on acquired cues. In line with the expectations, results showed microsaccadic rate inhibition and pupillary dilation depending on cognitive effort (number of acquired cues) prior to decision. These effects were moderated by affective priming. Aversivepriming strengthened pupillary and microsaccadic response to information processing effort.In general, results suggest that pupillary response is more biased by affective priming than microsaccadic rate. The results are discussed in the light of neuropsychological mechanisms of pupillary and microsaccadic behavior generation.

## Introduction & Background

Complex cognition requires effort and taxes elementary cognitive processes such as working memory and attention. Decision making with multiple cues is a good example of such effortful mental process. In addition, decision making is often performed under incidental emotional arousal elicited by external events. Eye tracking measures are well known as indicators of both mental effort and arousal (4, 2). In this paper, we investigate the sensitivity of microsaccadic and pupillary measures as indices of cognitive effort and emotional arousal during complex decision-making.

### Cognitive effort in decision making

Real-life situations such as inferring the selling or buying price of a car, or deciding which team is likely to win a volleyball match, are examples of decisions based on making probabilistic inferences. When making choices, decision makers often process multiple pieces of information, with some choices requiring information integration and others allowing for one-reason decision-making (5). Researchers have argued that in order to make such choices, people select decision strategies from a broad repertoire of methods, with two prominent examples being the Weighted Additive rule and Take The Best heuristic (6, 7).The complex Weighted Additive strategy integrates all available cues, whereas the simple heuristic Take The Best uses only one, the most important cue to make the choice. These strategies can be characterized by different level of cognitive effort that is needed in order to make a decision, with the simple heuristic requiring less effort than the complex strategy.

Research in neuroscience has elucidated neural mechanisms underlying the use of complex vs. simple strategies in decision making. Khader et al. (8) showed that the activity of the dorsolateral prefrontal cortex (DLPFC) reflects the number of cues needed to make a decision in a multi-attribute decision-making task. Venkatraman, Payne, Bettman, Luce & Huettel (9) showed the involvement of the prefrontal cortex (dorsomedial, dorsolateral and insular) in the use of complex, computationally demanding strategies. They also showed that the tendency to use simple strategies is associated with high activity of the ventral striatum (part of the dopaminergic neuromodulatory system) in response to gain prospects. In a similar vein, Oh-Descher, Beck, Ferrari, Sommer, & Egner (10) showed the involvement of the dopaminergic system (ventral tegmental area/substantia nigra region), as well as putamen and cerebellum in the use of simple strategies under time pressure.

Besides fMRI, psychophysiological methods have been successfully used to track early neural signatures of complex decision making. Wichary, Mata, & Rieskamp (11) showed the association of high skin conductance with selective use of information and reliance on the Take The Best heuristic. In EEG/ERP research, Wichary, Magnuski, Oleksy, & Brzezicka (12) showed that the pattern of P3 responses to the decision cues differs between the users of complex strategy and a simple heuristic. The P3 ERP component, together with skin conductance and pupil dilation, has been proposed as a physiological marker of the Locus Coeruleus-Norepinephrine System (LC-NE) activity (13). The LC is a noradrenergic brainstem nucleus with wide projections to the whole brain, including dense innervations to brain areas involved in selective attention processing e.g., prefrontal and parietal cortex, pulvinar nucleus and the superior colliculus (13, 14). The LC regulates arousal and is activated by a range of stressors, increasing NE availability at the target sites and thus modulating information processing throughout the brain. Given the close link between pupil dilation, information processing and the LC, it is viable to ask if changes in pupil size are associated with patterns of information processing in complex decision making. Indeed, Costa and Rudebeck (15) note that while LC activity and pupil size are correlated, the mechanism are far from clear.

### Pupil size, cognitive effort and arousal

Psychologically relevant stimuli can influence pupillary dilation as the result of a neural inhibitory mechanism on the parasympathetic oculomotor complex or Edinger–Westphal nucleus by LC-NE (16). Early research showed that pupil diameter increases with the difficulty of a cognitive task (17). Kahneman & Beatty (4) showed that during a short-term memory task, pupil diameter is a measure of the amount of material under active processing. They showed positive correlation between the length of a string of digits to be remembered and pupil size. Since then, it has been shown many times that pupil size reflects activities related to cognitive effort and attention (18, 19, 20).

Besides information processing, pupil dilation is implicated in responses to emotionally arousing stimuli. In the first study on this topic, Hess & Polt (21) showed the association between pupil dilation and emotional arousal. Pupils of both male and female observers dilated when they viewed images of half-naked members of the opposite sex. More recent research shows that, similarly to pleasant pictures, pupil size increases also when viewing unpleasant pictures, compared to neutral pictures (2).

### Microsaccades and information processing

Similar to pupil dilation, microsaccades can also be studied in the context of information processing. The human visual system is optimized for the detection of motion and change, possibly due to the constant refreshing of the retinal image, achieved as a result of fixational eye movements composed of microsaccades, drift and tremor (22). Microsaccades are rapid small-amplitude saccades with a rate of about one per second (23, 24), triggered by the Superior Colliculus (SC; 25, 26). Microsaccades enhance visual perception and, therefore, represent a fundamental motor process with a specific purpose for visual fixation. According to Engbert (23), while microsaccades primarily might be essential for visual perception at the physical level, they also undergo top-down modulation by high-level attentional processes. Siegenthaler, Costela, McCamy, Di Stasi, Otero-Millan, Sonderegger, Groner, Macknik, & Martinez-Conde (27) suggested that different levels of task difficulty modulate microsaccade parameters via changes in the intensity and shape of the rostral SC activity map. Fluctuations of SC activity at the rostral poles are thought to give rise to microsaccades during fixation.

There are several current studies that suggest a connection between microsaccadic generation and cognitive effort. Siegenthaler et al. (27) showed that microsaccade rate decreases and microsaccade magnitude increases with greater task difficulty. A possible explanation is that higher working memory load leads to difficulties in fixation execution, producing fewer microsaccades and decreased control over their magnitude (1). Microsaccadic suppression was also observed by Gao, Yan, & Sun (28) in different stages of arithmetic, non-visual task performance. The microsaccade rate in the calculation phase was two times smaller compared to the postcalculation phase. Similarly, Dalmaso, Castelli, Scatturin, & Galfano (3) showed that microsaccadic rate drops in the high-load condition of the memory task (200 – 400 *ms* after onset), compared to the low-load condition. Krejtz et al. (1) suggested that Inter-Trial Change in Pupil Dilation and microsaccade magnitude adequately discriminate task difficulty.

Chen, Martinez-Conde, Macknik, Bereshpolova, Swadlow, & Alonso (29) showed that increased task difficulty reduces interference caused by peripheral distracters, decreasing the likelihood that distracters will deviate the focus of attention. This may be why visual task difficulty modulates the activity of specific populations of neurons in the primary visual cortex.

Little is known about microsaccadic response to emotional state, although results presented by Kashihara, Okanoya, & Kawai (30) suggest that microsaccade dynamics can be influenced by exogenous emotional stimuli. In their study, event-related responses to unpleasant images significantly inhibited microsaccadic rate, compared to neutral, pleasant and scrambled pictures, in the 300-600 *ms* time window after onset.

### The Present Study

The aim of the present study was to explore the sensitivity of pupillary and microsaccadic activity in response to cognitive effort and emotional arousal during decision-making task. Taking into account the literature review, we hypothesized that increased pre-decisional information processing would be associated with cognitive effort resulting in pupil dilation and microsaccadic rate inhibition. Secondly, on an exploratory basis we tested whether emotional arousal manipulation moderates the relation between the eye-related measures and cognitive effort.

## Method

### Participants

Twenty-eight university students volunteered for the study. Participants were not rewarded for participating in the experiment. The study was approved by the SWPS University institutional review board. All participants had normal or corrected to normal vision. Data from seven participants were excluded due to high calibration error (over 0.55˚) or technical problems with completion of the procedure. The calibration scores for the final sample, on average, were below 0.5˚ on both horizontal and vertical axes. The final sample consisted of 21 participants (15 Females) with average age equals to 30.76 (*SD* = 7.52). Participants were randomly assigned to three experimental groups: aversive priming (*N* = 7), erotic priming (*N* = 6) or emotionally neutral priming (*N* = 8).

### Procedure

The experiment was conducted on a per-individual basis. After signing a consent form the eye tracking equipment was set up and calibrated with a 5-point calibration for each participant. The experimental procedure consisted of three phases: instruction, training (with three decisions trials), and the main phase (with 24 decision trials). Participants were instructed that they were going to make a choice between two diamonds based on cues describing the diamonds’ properties.

**Figure 1.**
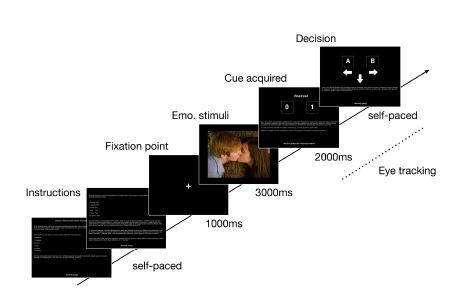
Experimental procedure scheme: instructions, fixation cross and trial elements comprised of: emotional picture, name of the cue, cue prevalence for diamond A or B, decision-making: picking diamond A, picking diamond B or take next cue (up to 6 cues).

Each trial started with a fixation cross presented for 1000 *ms,* followed immediately by an emotional stimulus (erotic, aversive or neutral) presented for 3000 *ms*. After emotional stimulus, the first cue was presented for 2000 *ms*. After the first cue presentation, the participant could decide whether to acquire the next cue (up to 6 cues) or make a choice between two diamonds A or B (see Figure 1). The eye tracking data were recorded during the cues presentation and decision making. After the experiment, participants were debriefed.

### The decision-making task

Participants decided which of two diamonds was more expensive based on acquired cues. The diamonds were represented by squares located side-by-side on a computer screen. The diamonds were described by up to six cues concerning their: size, clarity, shape, color, brilliance and proportions. The cue values were coded as 0 and 1, with 0 indicating a low value of the cue and 1 indicating a high value. After each cue, participants could make their choice by pressing the Left Arrow or Right Arrow key on the keyboard, or acquire the next cue by pressing the Down Arrow (see Figure 1). The average screen luminance for the cues and the decision-making part of the experimental procedure was 50 *lux*.

The cues were characterized by their validities: 0.706, 0.688, 0.667, 0.647, 0.625, 0.62 thus representing a compensatory task structure (see 31), where using complex strategies is most adaptive. The cue validities were conditional probabilities of making a correct choice, given that the cue discriminated between the alternatives (5). The validities were presented in the instruction, together with the information that the cues could be acquired sequentially in descending order of validity, from the best cue to the worst.

### Affective Priming

For affective priming we used a total of 75 stimuli, consisting of 25 pictures in each category (erotic, aversive, and neutral). All stimuli images were presented in color against a black background, at 1024×768 resolution. Erotic images were selected from Nencki Affective Picture System (32). The erotic images depicted opposite-sex couples kissing, hugging or engaged in sexual intercourse. Neutral and aversive images were chosen from the International Affective Picture System (33). The emotionally neutral images presented nonsexual objects e.g., boats, mugs, etc. The aversive images depicted strong negative and violent scenes e.g., mutilated bodies or images of suffering people.

We compared valence and arousal scores as well as luminance of the stimuli in three conditions. One-way ANOVA with experimental condition as a between-subject factor revealed a significant difference in arousal values of stimuli, *F*(2,72) = 231.37, *p* < 0.001, η^2^ = 0.87. Following pairwise comparisons with Tukey correction showed that all conditions differed significantly from each other in terms of arousal (see Table 1 for descriptive statistics). The ANOVA for valence also revealed significant difference between stimuli used in different conditions, *F*(2,72) = 471.29, *p* < 0.001, η^2^ = 0.93. Again, stimuli used in the study significantly differ between all three conditions (see Table 1 for descriptive statistics). The stimuli did not differ significantly between experimental conditions in terms of luminance, *F*(2,72) = 2.34, *p* = 0.10, η^2^ = 0.06 (see Table 1 for descriptive statistics).

**Table 1.** Valence and arousal ratings for used pictures picked from NAPS and IAPS databases. The presented ratings base on the information provided by the IAPS and NAPS stimuli sets’ authors.

Condition	Valence Mean (SD)	Arousal	Luminance (lx)
Erotic	6.42 (1.48)	4.84 (1.96)	107.52 (13.65)
Aversive	2.04 (1.41)	6.37 (2.49)	98.64 (18.20)
Neutral	5.08 (1.23)	2.68 (1.95)	107.33 (18.33)

Note: The valence scale ranges from 1 to 9, where: 1 – very negative emotions, to 9 - very positive emotions. The arousal scale ranges from 1 to 9, where: 1 – weak emotion, being emotionally unaroused, to 9 - strong emotion, being emotionally aroused.

### Apparatus

Eye movements were recorded binocularly by an SR Research EyeLink 1000 eye tracker running at a 1000 *Hz* sampling rate. During the recording each participant’s head was stabilized in a chin rest. The distance from the participant to the stimuli screen was 57 cm. The accuracy of the eye tracker reported by SR Research is 0,25˚ - 0,5˚ visual angle on average. The stimuli were presented on a 2200 LCD computer monitor (60 *Hz* refresh rate, 1024×768 resolution) connected to a standard PC. The experiment procedure was created with PsychoPy (34). The experimental room had no windows and ambient light remained constant during the entire experiment.

### Data Preprocessing

**Behavioral Measures.** Two major behavioral measures were collected during the course of the experiment and then analyzed: the number of acquired cues to make a decision and decision accuracy. Decision accuracy was a dichotomous measure consisting of 0 (wrong) and 1 (correct) values. The number of cues was treated as an indicator of decision-making cognitive strategy e.g., simple (single-cue) vs. complex (multi-cue). Making a decision after the first cue is a common indicator of a simple strategy, while taking the maximum and close-to-maximum possible number of cues is treated as an indicator of the complex strategy (7, 35, 36, 37, 38).

Note that more cognitive effort was needed to process a larger number of cues before a decision was made. Thus, number of acquired cues, for some analyses, was also treated as the measure of cognitive effort during the task.

**Pupil Dilation Measures.** Pupil diameter change estimates are traditionally related to cognitive load and cognitive effort (17, 4, 18, 20, 1). We employed two measures of pupil size changes which were demonstrated as reliable, the Inter-Trial Change in Pupil Dilation (see 39, 1). and the Low/High Index of Pupillary Activity (see 40, also compare 41, 1).

The Inter-Trial Change in Pupil Diameter (also named as Baseline Change in Pupil Diameter, BCPD) was computed using the smoothed pupil diameter signal subtracted from the baseline averaged smoothed pupil diameter obtained from the training trials of the experimental procedure. We assumed that the training trials did not induce cognitive effort or its extent was very small since the start of the entire experimental procedure. We decided to use inter-trial measure of pupil dilation change over intra-trial measure (e.g., using first 1000 *ms* as a baseline) based on literature review. The inter-trial measure was demonstrated as being more reliable and sensitive over intra-trial (see 1).

The Low/High Index of Pupillary Activity (LHIPA) is a novel measure of pupil activity during task performance, introduced first by Duchowski et al. (40). LHIPA is a ratio of low to high frequency, with high frequency response expected with increased cognitive effort, thus LHIPA is expected to decrease with increased cognitive effort. LHIPA was shown previously to discriminate task difficulty vis-à-vis cognitive load in a series of experiments where participants performed easy and difficult mental arithmetic tasks with fixed gaze, an *nBack* *task*, or easy and difficult eye typing with unrestricted eye movements (40). For details on implementation of LHIPA see (40, 41).

**Microsaccade Measures.** Following the literature on microsaccadic responses to cognitive effort (26, 27, 1), we focused on microsaccade magnitude (MS Magnitude) and rate (MS Rate) as dependent variables. Both have been demonstrated to be reliable measures sensitive to task difficulty and cognitive effort (26, 27, 1). Microsaccades were detected using the algorithm described in detail by Krejtz et al. (1) and based on Engbert & Kliegl (42). Before detecting microsaccades blinks were removed from raw gaze data, then, following Duchowski et al. (43), both left and right gaze points were averaged, i.e., (x(t), y(t)) = ([xl(t) + xr(t)]/2, [yl(t) + yr(t)]/2) which was used as a source data for fixation detection. The microsaccades were detected within each fixation during looking at cues and decision-making screens (see Figure 1). For more detailed description of the algorithm refer to Krejtz et al. (1) and Duchowski et al. (41).

**Table 2.** Proportion of cues used before decision in different experimental conditions.

	Number of cues				
Condition	1	2	3	4	5
Neutral	0.41	0.10	0.06	0.08	0.35
Aversive	0.54	0.12	0.04	0.01	0.29
Erotic	0.56	0.10	0.07	0.02	0.25
Overall	0.49	0.11	0.06	0.04	0.30

**Implementation of Eye-Movement Measures.** In order to capture the changes in eye movements over the course of each trial, we calculated differential measures for pupillary as well as microsaccadic estimates. These measures were calculated for each trial as the difference between the estimate during the last and first cue used by each participant. This resulted in ∆BCPD, ∆IPA, ∆LHIPA, ∆MS Rate, and ∆MS Magnitude measures which were implemented into the statistical model analyses as dependent variables. The interpretation of such measures is relatively straightforward. For example, negative values of ∆BCPD reflect the fact that Inter-Trial Pupil Diameter constricted over the time course of the trial while positive values mean that it dilated.

## Results

In order to test our hypotheses, first we determined the number of cues acquired by each participant before making each choice in each experimental condition. Participants could use up to 6 cues. Since the frequency of acquiring all six cues was minimal, we focused our analyses on 5 cues. The distribution of acquired cues was tested with the χ^2^ tests for the goodness of fit, separately for each experimental condition.

Before running the hypotheses’ tests for microsaccades, we checked if the detected microsaccades follow the main sequence (the relation between microsaccadic velocity and magnitude). The main sequence test was performed with the use of a linear regression model.

To test the hypotheses related to pupillary and microsaccadic measures, nested linear mixed models (LMM) were estimated with Maximum Likelihood method. Due to the nested nature of the data the tested models were on two levels: experimental condition and participant, constituting random effects. All present models included also two fixed effects: experimental condition as between-subject fixed factor and the number of acquired cues as a within-subjects fixed factor. All statistical analyses were performed using the R language for statistical computing^44^ and the LMM models were fitted with *lme4* R library.

### Behavioral Responses

**Number of acquired cues.** In line with expectations, the distribution of cues used by participants to make decisions suggested the use of two vastly different strategies. Out of all choices, 49% were based on only one cue, strongly suggesting the use of the Take The Best heuristic, while 30% were based on 5 cues suggesting the use of the complex Weighted Additive rule (see Table 2 for detailed distribution values). The proportion test comparing cues’ distribution to distribution of equal proportions was statistically significant, χ^2^(5) = 360.20, p < 0.001. Similar distributions of the number of acquired cues was observed for each experimental condition, see Table 2. All of the distributions were statistically different from the flat distribution (for neutral condition, χ^2^(5) = 131.18, p < 0.001, for averse condition, χ^2^(5) = 178.71, p < 0.001, and for the erotic condition, χ^2^(5) = 164.46, p < 0.001).

### Pupil Diameter

We hypothesized that increase in pupil size and the changes in pupillary activity index are sensitive to emotional arousal and pre-decisional cognitive effort. We tested these hypotheses with LMM models with the experimental condition and the number of acquired cues as fixed factors. The model included also the interaction term of these factors. In the first analysis, we treated the Inter-Trial Pupil Dilation difference between the last and first acquired cue (∆BCPD) as the dependent variable. In the second analysis, the Low/High Index of Pupillary Activity difference between last and first cue (∆LHIPA) was the dependent variable.

**Inter-Trial Pupil Dilation (BCPD).** Before estimating the LMMs, a zero-order Pearson correlation test was performed between the number of acquired cues and the difference in Inter-Trial Pupil Diameter during the last and first cue (∆BCPD). The correlation was moderate but only marginally significant, *r* = 0.412, *t*(19) = 1.970, *p* = 0.064.

The null model of LMM analysis showed satisfying indices of model fit with *pseudo-R^2^* *(total)* = 0.15. The average for ∆BCPD (model intercept) was 104.897 with ample source of variance at both levels of analyses (*s^2^* = 16542.80 for participants’ level and *s^2^* = 790.80 for experimental conditions’ level).

The full model (with *pseudo-R^2^* *(total)* = 0.19) was significantly different from the null model, χ^2^(5) = 37.167, *p* < 0.001. The fixed effect of number of acquired cues was statistically significant, *F*(1, 112.634) = 12.174, *p* < 0.001. The model coefficients showed that the number of acquired cues significantly predicts pupil dilation, *b* = 37.00, *SE* = 15.90, *t*(101.76) = 2.327, *p* = 0.022. This relation was moderated by experimental condition.

**Figure 2.**
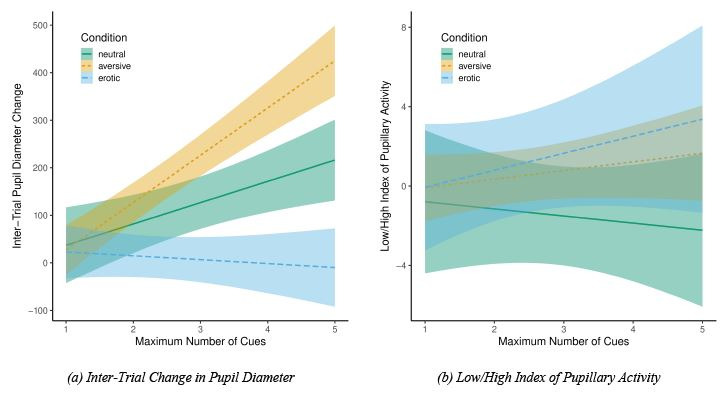
Pupillary measures (Inter-Trial Change in Pupil Diameter and Low/High Index of Pupillary Activity) in response to emotional arousal and cognitive effort (number of acquired cues). *Note: gray areas denote the regression lines’ confidence intervals*.

The interaction between the number of acquired cues and experimental condition was significant, *F*(2, 111.891) = 11.871, *p* < 0.001. In comparison to neutral condition, in the aversive condition, the number of acquired cues predicted pupil dilation, *b* = 61.79, *SE* = 24.23, *t*(97.92) = 2.550, *p* = 0.010, but in the erotic condition the number of acquired cues predicted pupil constriction, *b* = -65.86, *SE* = 24.58, *t*(124.21) = 2.679, *p* = 0.008, see Figure 2(a).

**Low/High Index of Pupillary Activity (LHIPA).** We started the analyses by testing the relation between the number of acquired cues and the change in Low/High Index of Pupillary Activity (∆LHIPA) from the first cue to the last with zero-order correlation test. The test showed that the relation is close to zero, *r* < 0.001, *t*(19) = 0.002, *p* = 0.998.

The LMM analyses were started with the null model with random effects of experimental condition and the number of acquired cues. The null model revealed *pseudo-R^2^* *(total)* = 0.04. The intercept of null model was not significantly different from zero, *b* = - 0.084, *SE* = 0.907, *t*(20.815) = - 0.093, *p* = 0.927, with the *s^2^* = 8.453 at participants’ level and *s^2^* = 0.000 at experimental condition level. Taking this into account, not surprisingly the full model was not significantly different from null model, χ^2^(5) = 6.547, *p* = 0.257. It showed also no significant effects of the number of acquired cues, *F*(1, 93.305) = 2.205, *p* = 0.141, experimental condition, *F*(2, 36.947) = 0.374, *p* = 0.690 nor interaction term, *F*(2, 92.600) = 1.9460, *p* = 0. 1486, see Figure 2(b).

### Microsaccades

The analyses of microsaccadic response to cognitive effort related to the number of acquired cues, and to emotional condition started with a check of the microsaccadic main sequence, the expected pattern of a linear relationship between microsaccade magnitude and peak velocity (see 27). That analysis was followed by two separate Linear Mixed Models (LMM) to check the sensitivity of the two major microsaccadic characteristics (magnitude and rate) to the experimental condition and the number of acquired cues.

**Figure 3.**
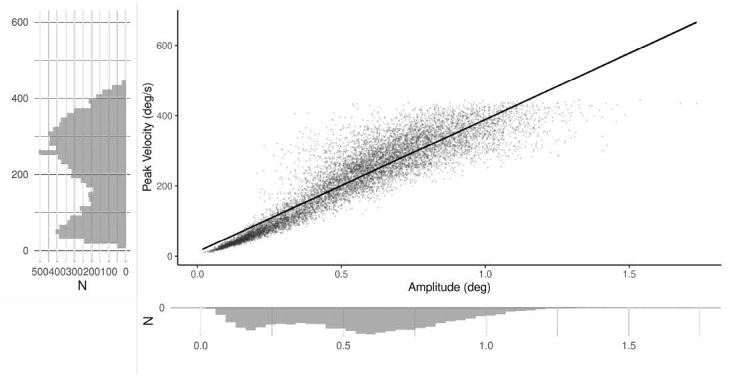
Microsaccade main sequence - general relation between microsaccade magnitude and peak velocity. The line represents the estimated linear model for the relation, while dots represent all detected microsaccade.

**Main Sequence Validation.** To check the main sequence relation between microsaccade peak velocity and amplitude we ran a simple regression with the microsaccade magnitude treated as a predictor and peak velocity as a response variable. The analysis showed that the model explained over 83% of the variance, *F*(1,10665) = 55260.00, *p* < 0.001, *R^2^* = 0.838. Microsaccade magnitude strongly predicts microsaccade peak velocity, *b* = 376.37, *SE* = 1.60, *t*(10665) = 235.08, *p* < 0.001, see Figure 3. The intercept of the model was also statistically significant, *b* = 12.91, *SE* = 0.99, *t*(10665) = 13.04, *p* < 0.001. This relation is highly consistent with previous literature (see e.g., 27, 1).

**Microsaccade Rate.** Before running the LMMs, we checked the correlation between the number of acquired cues and the microsaccade rate. The analyses revealed moderate negative, however not significant, relation between the variables, r = -0.374, t(19) = 1.756, p = 0.095. We then ran the LMM analysis with microsaccade rate as the dependent variable and the experimental condition and the number of acquired cues as fixed factors. The null model with random effects and intercept only, revealed pseudo-R2 (total) = 0.07. The intercept was significantly different from zero, b = -0.271, SE = 0.112, t(18.328) = 2.412, p = 0.027 with random effects variance at participants’ level (s^2^ = 0.169) and no variance at experimental condition level (s^2^ < 0.001).

**Figure 4.**
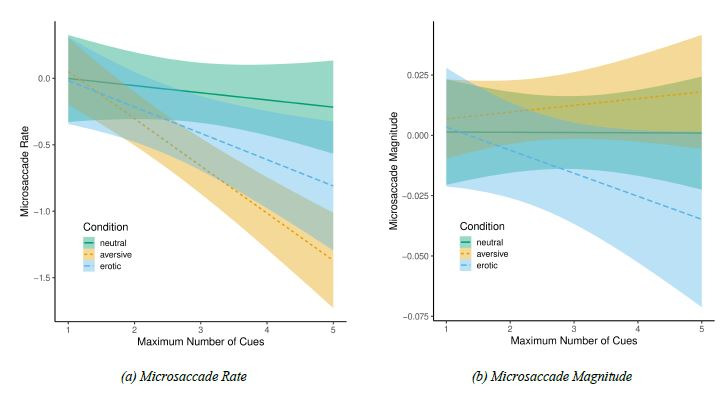
Microsaccadic rate and magnitude in response to emotional arousal and cognitive effort (number of acquired cues). *Note: gray areas denote the regression lines’ confidence intervals*.

Nevertheless, the full model with both main effects and interaction term was significantly different from the null model, χ^2^(5) = 26.631, p < 0.001, with pseudo-R² (total) = 0.08. The model revealed that the experimental condition did not predict microsaccade rate, F(2, 472) = 0.796, p = 0.452. The effect of acquired cues was significant, F(1, 472) = 27.462, p < 0.001. The increase in number of acquired cues significantly predicted microsaccade rate decrease, b = - 0.355, SE = 0.069, t(472) = 5.182, p < 0.001. Also, the interaction between fixed factors was significant, F(2, 472) = 5.546, p = 0.004. The model coefficients showed that the slope of the relation between the number of acquired cues and microsaccade rate was significantly steeper in the aversive condition than in the neutral condition, b = - 0.302, SE = 0.091, t(472) = 3.325, p < 0.001. The erotic condition did not differ significantly from neutral nor from aversive conditions, see Figure 4(a).

**Microsaccade Magnitude.** Analogous analyses for microsaccade magnitude yielded no significant effects, see Figure 4(b).

The full model microsaccade magnitude with both main effects and interaction term was not significantly different from the null model, χ^2^(5) = 6.681, *p* = 0.245, with *pseudo-R² (total)* = 0.02. None of the effects in the full model were significant either: the experimental condition effect (*F*(2, 0) = 0.130, *p* = 1), the effect of acquired cues (*F*(1, 466) = 0.714, *p* = 0.399), and the interaction term (*F*(2, 466) = 1.719, *p* = 0.180).

## General Discussion

The present study investigated sensitivity of pupillary and microsaccadic measures to cognitive effort and arousal during complex (multi-attribute) decision making. It is one of the first studies to show the joint impact of these factors on microsaccade rate dynamics. First, we assumed that affective priming would influence participants’ emotional arousal during decision making. Second, we expected that the number of cues acquired prior to decision would increase participants’ cognitive effort. The hypotheses predicted that pupillary and microsaccadic rate would reflect these manipulations.

Behavioral results from the present experiment showed that participants varied in the extent of pre-decisional information processing, with some choices based on a single cue, suggesting the use of the simple Take The Best heuristic and other choices based on several cues, suggesting the use of complex decision rules (7, 35).

### Pupil size vs. cognitive effort and affective priming

The number of cues acquired prior to decision, together with the affective priming, influenced pupil size. In the aversive and neutral conditions, pupil size linearly increased. On the other hand, in the erotic condition, pupil size did not react to cognitive effort. These results suggest that affective priming with highly arousing aversive stimuli makes pupil dilation particularly sensitive to cognitive load and, on the other hand, priming with highly arousing positive stimuli makes it less sensitive to cognitive load.

These results are consistent with theories and research on cognitive control adaptation and the aversive nature of cognitive control. Cognitively demanding situations (e.g., cognitive conflict, cognitive load) are perceived as aversive and result in cognitive control adaptation, showing increased control which allow to counteract a deterioration of performance due to demanding conditions (45, 46, 47). Studies on affective modulation of cognitive control (48, 49, 50) also show that affective priming with negative stimuli increases cognitive control and positive, rewarding stimulation decreases or entirely cancels the impact of task demands on control adaptation.

On the other hand, these different influences of positive vs. negative arousing stimuli on pupil size seem incosistent with results showing that both positive and negative arousing stimuli elicit similar pupil dilations which substantially differ from the responses elicited by neutral stimuli (2, 51). However, those studies are not easily comparable with ours, because they only involve pupil responses to affective stimuli, but do not involve responses to cognitive load and thus are mute about control adaptation in complex cognitive tasks.

The neural substrate of the relation between positive vs. negative affective priming and pupil response to cognitive load can be explained by the growing body of evidence showing that cognitive control is primarily subserved by the anterior cingulate cortex (ACC; 52, 47), which responds to pain, anxiety, cognitive effort and other demanding bodily states. The ACC is tightly reciprocally linked with the Locus Coeruleus (LC) and this loop is a postulated neural substrate of cognitive control adaptation (13, 20). Thus, one possible scenario for control adaptation is that under conditions demanding cognitive control (e.g., high cognitive load) ACC activates LC, which results in increased norepinephrine (NE) output in the cortex (and in other parts of the brain). The effect of increased NE output is an increase in information processing gain, which results in prioritized processing of stimuli that are most relevant to task performance. LC activity is tightly linked to pupil size changes (53, 54, 55), therefore control adaptation can be observed as increases in pupil size in cognitively demanding tasks. In this perspective, the effect of erotic priming can be explained by the fact that ACC activity is modulated by the brain reward systems, primarily the dopaminergic and the opioid system, which is supported by the presence of numerous dopamine and opioid receptors in ACC (56, 57).

### Microsaccades vs. cognitive effort and affective priming

The analyses revealed that the number of acquired cues and affective priming influence the microsaccade rate but not microsaccade magnitude. Microsaccade rate decreased linearly with the number of acquired cues. This relation was the most pronounced in the aversive priming and the least in the neutral affective priming condition. In the erotic condition, this relationship did not differ neither in the neutral nor aversive condition. The pattern of results suggests that microsaccade rate is less sensitive to arousal than pupil dilation, at least in the context of complex decision-making task.

In general, presented results are consistent with current literature showing that microsaccade rate is a valid and reliable metric of cognitive effort (3, 28, 1, 27). They are consistent also with research concerning relationship of arousal and microsaccades dynamics demonstrating that microsaccadic activity can be modulated by exogenous emotional stimuli (see 30).

The mechanism of the impact of cognitive effort and arousal on microsaccade rate is less understood than pupillary response, however it is likely that LC activity is also involved here. Microsaccades are generated by changes in neural activity in the rostral parts of Superior Colliculus (SC; 58) and SC activity is functionally linked with LC activity, as shown by Joshi et al. (54) in the context of pupil dilation. This is also supported by anatomical connections between LC and SC as shown by Li et al. (59). It is also possible that emotional arousal activates the Basal Ganglia-BrainStem system (BG-BS), indirectly resulting in microsaccade suppression. Neuron clusters in the SC receive transmitter-specific afferents from the pedunculopontine tegmental nucleus and from GABAergic cells in the substantia nigra, that impose a tonic inhibition on the Superior Colliculus (60). The pedunculopontine nucleus is connected to the BG-BS system, which is responsible for the manifestation of volitionally-directed and emotionally-triggered motor behavior consolidation (61).

These functional and anatomical relations underlie the role of the Locus Coeruleus-Norepinephrine system in not only generating pupillary responses to cognitive effort and emotional arousal but possibly also are indicative of microsaccadic response. Our study shows that the joint impact of these factors can be observed in the context of a complex decision-making task. As in previous research, we show that changes in pupil size and microsaccade rate reflect cognitive effort. Moreover, our results suggest that microsaccade rate dynamics reflect the impact of emotional arousal better than pupil size dynamics, which is strongly influenced by affective stimulus valence. Therefore, microsaccade rate is a good candidate for an index of both cognitive effort and emotional arousal in future studies on these topics.

### Ethics and Conflict of Interest

The author(s) declare(s) that the contents of the article are in agreement with the ethics described in http://biblio.unibe.ch/portale/elibrary/BOP/jemr/ethics.html and that there is no conflict of interest regarding the publication of this paper. The authors declare that there is no conflict of interest regarding the publication of this paper.

### Acknowledgments

This work is partly supported in part by Marie Skłodowska-Curie Fellowship (project no. 791181) granted to Szymon Wichary.

Open access of this article was financed by the Ministry of Science and Higher Education in Poland under the 2019-2022 program „Regional Initiative of Excellence", project number 012 / RID / 2018/19.

We thank reviewers for their suggestions for improvement.
